# Transcatheter Closure of Perimembranous Ventricular Septal Defects in Children using a Wire-Drifting Technique

**DOI:** 10.6061/clinics/2018/e371

**Published:** 2018-11-16

**Authors:** Lu He, Ge-Sheng Cheng, Yu-Shun Zhang, Xu-Mei He, Xing-Ye Wang, Ya-Juan Du

**Affiliations:** Department of Structural Heart Disease, The First Affiliated Hospital of Xi'an Jiaotong University, Shaanxi Province, China

**Keywords:** Ventricular Septal Defect, Transcatheter, Wire-Drifting, Atrioventricular Block, Technique

## Abstract

**OBJECTIVE:**

Explore the feasibility and safety of transcatheter closure of perimembranous ventricular septal defects using a wire-drifting technique (WT) in children.

**METHODS:**

We retrospectively analyzed 121 pediatric patients diagnosed with perimembranous ventricular septal defects who underwent interventional treatment at the First Affiliated Hospital of Xi'an Jiaotong University from Dec 2011 to Dec 2014. Based on the method used for arteriovenous loop establishment during the procedure, the patients were divided into a conventional technique (CT) group and a WT group.

**RESULTS:**

In total, 51 of the 53 patients (96.2%) in the CT group and 66 of the 68 patients (97.1%) in the WT group achieved procedural success, with no significant difference between the two groups (*p*>0.05). The CT group showed a nonsignificantly higher one-time success rate of arteriovenous loop establishment (94.3% *vs*. 91.2%, *p>*0.05). The procedure time was 46.0 (14.0) min and 46.5 (10.0) min in the CT and WT groups, respectively. The CT procedure was discontinued in the 2 cases (3.8%) of intraprocedural atrioventricular block in the CT group. In the one case (1.9%) of postprocedural atrioventricular block in the CT group, a permanent pacemaker was implanted to resolve third-degree atrioventricular block three months after the procedure. In the WT group, no cases of intraprocedural atrioventricular block occurred, and one case (1.5%) of postprocedural atrioventricular block occurred. In this case, intravenous dexamethasone injection for three days returned the sinus rhythm to normal. Aggravated mild to moderate tricuspid regurgitation was observed in 2 patients (3.8%) in the CT group during the 2-year follow-up period; aggravated tricuspid regurgitation was not observed in the WT group. During the 2-year follow-up period, there was no evidence of residual shunting in either group.

**CONCLUSION:**

Transcatheter closure of perimembranous ventricular septal defects with the WT is safe and effective in children.

## INTRODUCTION

Since 2002, when Hijazi 1 first used the new Amplatzer occluder to treat perimembranous ventricular septal defects (PmVSD) and achieved success, transcatheter closure has become an effective method for the treatment of PmVSD 2,3. However, the transcatheter closure of ventricular septal defects (VSD) is not currently approved in the United States because of unacceptably high rates of heart block (2.9%–5.7%), both post-procedural and late-onset 3-5. Nevertheless, this method is widely used in some countries, such as China and India, with a low rate of post-procedure heart block using modified nitinol shape-memory alloy devices made in China 6,7. However, for cases with multiple interventional treatments, heart block during and after the procedure is often reported and appears serious 8. According to the literature, the rate of complete atrioventricular block (AVB) during the procedure is 1.2% 8; in the early postoperative period, the rate of complete AVB is 0.1%–4% by surgery but 1.0%–6.4% by transcatheter closure 9. There is sometimes a need to implant a permanent pacemaker, particularly in children 4,10. There are many causes of heart block during or after intervention occlusion. Aside from occluder factors, one of the most important causes of heart block occurs when the arteriovenous loop is established during the procedure; repetitive stimulation of the peripheral tissue with the catheter and guidewire can lead to left or right bundle branch block, intraventricular block, and even AVB 8. Due to the small area of the membranous parts of the interventricular septum and surroundings, the conduction system is situated close to the edge of the defect, which is more prevalent in children. In this study, to reduce stimulation from the catheter and guidewire to the peripheral tissues, we used a wire-drifting technique (WT) to establish an arteriovenous loop. The aim of this study was to explore the feasibility and safety of the WT in the transcatheter closure of PmVSD in children.

## MATERIALS AND METHODS

### Study population

From December 2011 to December 2014, 149 children received intervention therapy for PmVSD at the First Affiliated Hospital of Xi'an Jiaotong University. By reviewing surgical records, we excluded 28 cases of mixed use of the conventional technique (CT) and a WT to establish an arteriovenous loop. A total of 121 children were retrospectively analyzed.

The parents of the children provided informed written consent for the procedure. The local ethics committee approved the surgery. The patients included 55 males and 66 females with an age range of 3 to 14 (median 4) years. All patients were diagnosed by clinical examination and transthoracic echocardiography (TTE). Among the patients, 3 patients had concomitant patent ductus arteriosus (PDA), 2 patients had atrial septal defects (ASD), and 1 patient had a corrected transposition of great vessels (type SLL). The inclusion criteria for closure were as follows: ① age 3–18 years; ② maximum diameter ≤12 mm by TTE; ③ defect located at the 9 to 11 o’clock positions of an analog clock in the short-axis parasternal view; ④ left-to-right shunt; and ⑤ pulmonary pressure <70 mmHg by TTE^6^. Based on whether the WT was used, the patients were divided into the CT group (53 patients; the conventional method was used to establish an arteriovenous loop) and the WT group (68 patients; the WT was used to establish an arteriovenous loop). The exclusion criteria were as follows: ① less than 3 years or more than 18 years old; ② aortic valve prolapse; ③ moderate to substantial aortic regurgitation; ④ moderate to substantial tricuspid regurgitation; ⑤ right-to-left shunt or bidirectional shunt; ⑥ NYHA III–IV; and ⑦ residual defects after surgery.

### Echocardiographic protocols and definitions

The GE-ViVid-E9 color Doppler ultrasound system (General Electric Corporation, Norfolk, Virginia) equipped with a 2–4 MHz transducer was used to perform TTE. All patients underwent TTE before the transcatheter procedure. The PmVSD was evaluated with TTE in the long axis parasternal view, apical 5-chamber view, and short-axis parasternal view. The pre-procedure TTE measurement of VSD size was 8.0 (3.0) mm, and the ventricular septal rim below the aortic valve was 3.0 (0.5) mm.

### Description of the modified double-disk occluder

This study used a modified self-expandable double-disk occluder (ShangHai Shape Memory Alloy, Shanghai, China) that was based on the Amplatzer occlude 6,. There were 3–5 polyester patches in each disk, which guaranteed the sealing of the occluder. Two subtypes of double-disk occluders were used in this study: symmetric and asymmetric occluders. The left disk was symmetric in the symmetric occluder. The diameter of the disk was 4 mm larger than that of the waist. In the asymmetric occluder, the flange of the left ventricular (LV) disk facing the aortic valve was the same size as the waist, while the flange of the disk on the opposite side of the aortic valve was 6 mm larger than the waist. The right ventricular (RV) disc of the asymmetric occluder was the same as that of the symmetric occluder. The waist length of the occluder was between 2.5 mm and 4 mm, depending on the diameter of the disk. The asymmetric device was used for patients with a <2 mm rim under the aortic valve; for patients with a >2 mm rim under the aortic valve, the symmetric device was used. The delivery system consisted of a sheath (5–9 Fr), dilator, pusher, cable, loader, and pin vise.

### Catheterization and device implantation technique

All the operations were performed by the same surgeon and first assistant. If the patient had a concomitant PDA or ASD, the VSD was first resolved before addressing the PDA or ASD. All patients received antibiotics before the procedure. The procedure was performed under 2% lidocaine local anesthesia for patients aged 7 years and above, while general anesthesia with ketamine was used in younger children. Fluoroscopy was used to guide the device implantation only. The femoral vein and artery were accessed, and intravenous heparin (80–100 IU/kg) was administered. Routine right and left heart catheterization was performed to assess the degree of shunting and to evaluate the pulmonary vascular resistance. Hemodynamic parameters, including pulmonary arterial, aortic, atrial, and ventricular pressures, were recorded. Oximetry values were measured in the cardiac chambers and vessels, and the pulmonary-to-systemic flow ratio (Qp/Qs) was calculated based on these values. Angiography in a single plane (45°–60° left anterior oblique/10°–25° cranial view) was performed to define the location and size of the PmVSD. An angiogram of the ascending aorta was also performed in the 50° left atrial oblique view to check for aortic insufficiency. The size of the defect and its relationship to the aorta were confirmed.

**Figure 1:**
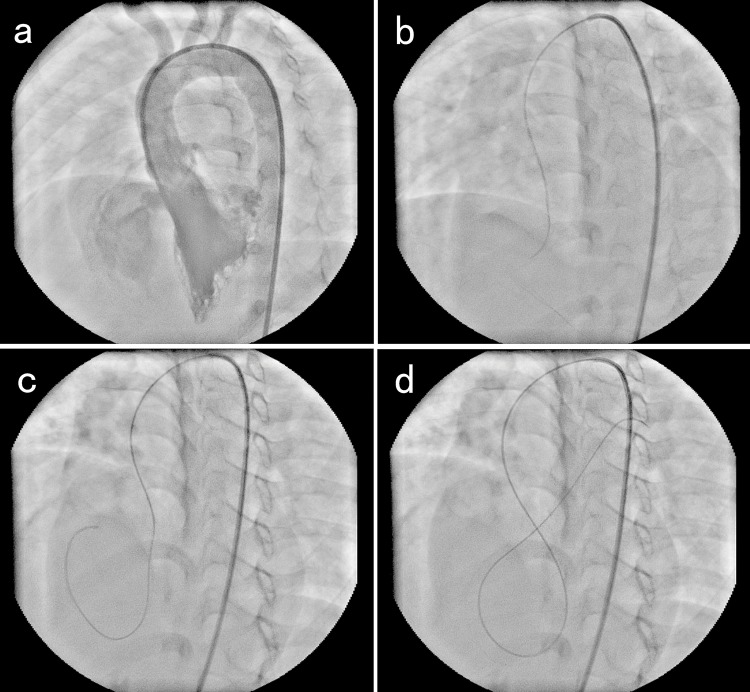
The process of the wire-drifting technique a: Left ventricular angiography showing the PmVSD. The distance from the aortic valve to the upper edge of the defect was approximately 3 mm, and the diameter was approximately 6 mm. b: The catheter was in the aortic arch, and the guidewire was advanced along the catheter into the left ventricle. c: The guidewire drifted with the direction of flow through the defect to the right ventricle. d: The guidewire in the right ventricle drifted to the distal end of the pulmonary artery with the direction of flow.

The standard PmVSD closure technique that was used has been previously described 15,16. The procedure was discontinued when second or third-degree AVB occurred. Dexamethasone (2 mg/kg) was immediately administered to the patient, and a temporary pacemaker was inserted when Mobitz type II AVB or complete AVB occurred during the procedure. The largest difference between the CT and WT groups was the method used to establish the arteriovenous loop. Depending on the morphology of the PmVSD, a 5 Fr Judkins right coronary, partially cut pigtail or 3DRC catheter was selected to guide the soft-tipped wire, which was pointing in different directions. We employed two different methods to cross the interventricular septum. When establishing the arteriovenous loop in the CT group, the inlet (LV surface) of the VSD was hooked directly to the catheter. After making sure the tip of catheter was at the inlet of the VSD, the guidewire was pushed gently out of the outlet (RV surface) of the VSD. Then, the guidewire drifted with the direction of flow to the pulmonary artery or superior/inferior vena cava. When establishing the arteriovenous loop in the WT group, the catheter was inserted into the ascending aorta and aortic arch while ensuring that the catheter was not inserted into the LV. Taking advantage of the aortic valve opening, the guidewire was then inserted into the LV. The catheter was rotated and adjusted up and down to facilitate exploration of the inlet of the VSD with the wire. Then, the wire drifted with the direction of flow through the VSD to the RV and pulmonary artery or through the right atrioventricular valve to the superior/inferior vena cava (Figure 1). The following procedural steps were reported in the literature 15,16. The sizes of the devices were selected to be 1–3 mm larger than the diameter of the defect, as assessed by TTE or angiography.

### Follow-up protocol

After the procedure, single-dose antibiotic therapy was administered intravenously to prevent infective endocarditis. Electrocardiographic monitoring was used for 1 day, and then electrocardiography was performed every day until discharge. Patients were commonly discharged 5 days after the procedure. Aspirin (3 mg/kg daily) was administered for 6 months to all patients after the procedure. A clinical examination, TTE (used to assess residual shunts, device position and relation to aortic and tricuspid valves, valvular regurgitation, and LV diameters), and chest radiography were performed before discharge; at 1, 3, 6, 12, and 24 months after device implantation; and yearly thereafter.

### Statistical analysis

Data analysis was performed using SPSS version 19.0 (Statistical Package for Social Sciences, version 19.0 for Windows, SPSS, Chicago, Illinois). Data for normally distributed quantitative variables were expressed as the means±standard deviation (SD). Differences in means for continuous variables were compared using Student's *t*-test. For non-normally distributed variables, the data were presented as the medians and interquartile range (IQR). Differences in the mean for non-normally distributed variables were compared using the Mann-Whitney U test. Categorical data were summarized by ratios and percentages. The χ^2^ test or Fisher's exact test was used for two-group comparisons. A *p*-value of <0.05 was considered statistically significant.

## RESULTS

### Patient characteristics

There were no significant differences in age, sex, weight or other baseline data between the two groups. In the CT group, the TTE measurement of defect size was 8.0 (4.0) mm, and the ventricular septal rim below the aortic valve was 3.0 (1.5) mm; 38 patients (71.7%) had a membranous ventricular septal aneurysm (MVSA), 3 patients (5.7%) had minor aortic regurgitation, and 2 patients (3.8%) had minor tricuspid regurgitation. In the WT group, the TTE measurement of defect size was 8.0 (1.8) mm, and the ventricular septal rim below the aortic valve was 3.0 (0.7) mm; 49 patients (72.1%) had MVSA, 4 patients (5.9%) had minor aortic regurgitation, and 2 patients (2.9%) had minor tricuspid regurgitation. The specific parameters evaluated are listed in Table 1.

### Procedural data

Among the 53 patients in the CT group, 51 patients achieved procedural success; the success rate was 96.2%. The procedure was aborted in two patients because a third-degree AVB occurred when the catheter entered the defect. Dexamethasone (2 mg/kg) was immediately administered to these two patients, and a temporary pacemaker was subsequently inserted. Continuous electrocardiographic monitoring was performed 24 hours after the procedure. The 2 patients with third-degree AVB were converted to sinus rhythm approximately 12 hours after the procedure, and the temporary pacemaker was removed. Among the 68 patients in the WT group, 66 patients achieved procedural success; the success rate was 97.1%. In the 2 unsuccessful patients, the catheter failed to pass the defect.

In the CT group, the average operation time was 46.0 (14.0) min, and the average fluoroscopy time was 24.0 (8.0) min. In the WT group, the average operation time was 46.5 10 min, and the average fluoroscopy time was 22.5 (5.0) min. There were no statistically significant differences in procedure time or fluoroscopy time between the two groups.

The CT group showed a higher one-time success rate in arteriovenous loop establishment (94.3% *vs*. 91.2%). In the CT group, arteriovenous loop establishment was successful on the first try for 50 patients. In the 3 patients without first-time success in establishing the loop, when the initial arteriovenous loop was established, it was found that the guidewire rode across the tricuspid chordae tendineae, indicating that the arteriovenous loop should be established again. In the WT group, the arteriovenous loop was successfully established on the first try in 62 cases. In the 6 patients without first-time success in establishing the loop, the outlet of defect was multi-crevassed, and the guidewire drifted through the minor crevasse into the RV; therefore, the position of the guidewire required readjustment to establish the arteriovenous loop.

During the procedure, the incidence of AVB in the WT group was lower than that in the CT group (3.8% *vs*. 0%). In the CT group, 2 cases (3.8%) of third-degree AVB occurred; therefore, the procedure was discontinued. No AVB occurred in the WT group. There was no statistically significant difference in the incidence of post-procedure AVB between the two groups (1.9% *vs*. 1.5%). Third-degree AVB occurred in one patient (1.9%) within 3 days after the procedure. After the intravenous injection of dexamethasone for 7 days, the heart rhythm of the patient was still third-degree AVB. After 3 months of follow-up, the heart rhythm was still third-degree AVB; a permanent pacemaker was implanted. In the WT group, no cases of AVB occurred during the procedure; one case (1.5%) of AVB occurred after the procedure and was reverted to sinus rhythm by the intravenous injection of dexamethasone for 3 days.

Aggravated mild-to-moderate tricuspid regurgitation was observed in 2 patients (3.8%) in the CT group at the 2-year follow-up. There was no occurrence of aggravated tricuspid regurgitation in the WT group. The incidence of aggravated tricuspid regurgitation post-procedure in the WT group was lower than that in the CT group (0% *vs*. 3.8%) (Table 2).

## DISCUSSION

The longest step in the transcatheter closure of PmVSD is the establishment of the arteriovenous loop. Ventricular tachycardia, supraventricular tachycardia and even AVB may occur during the procedure. These arrhythmias are strongly related to the stimulation of the endocardium by the catheter and guidewire, especially in the establishment of the arteriovenous loop 13.

The reported occurrence of AVB is approximately 0.1%–4% with surgery but 1.0%–6.4% with transcatheter closure 9. In our study, the incidence of AVB during the procedure was 3.8% in the CT group and 0% in the WT group. The exact underlying mechanism of AVB remains speculative, but the occurrence of an AVB in the procedure when the arteriovenous loop was created suggests that the system of conduction was injurious. By definition, the lower edge of the PmVSD is the atrioventricular bundle and its branches. Milo et al. 17 reported that the distance from the atrioventricular bundle to the edge of the defect was only 2-4 mm, and the left and right bundle branches could be wrapped in the residual fibrous tissue at the edge of the defect. Based on the process of transcatheter closure of the PmVSD, AVB occurrence during the procedure may be related to the following events: when the catheter seeks the inlet of the defect; when the catheter or guidewire goes through the defect; and when the catheter or guidewire goes through the defect into the RV and the pulmonary artery or vena cava. The catheter or guidewire may stimulate the left or right bundle branch and cause left or right bundle branch block, ventricular block, or even AVB 18. Especially in pediatric patients, the myocardial fibers are relatively weak, and the atrioventricular bundles are small. When the myocardium encounters stimulation, friction or extrusion, the myocardial tissues are more prone to edema in a larger area, which is more likely to involve the heart conduction system; therefore, pediatric patients are more likely to experience heart block during or after the procedure. When the arteriovenous loop is established by the conventional method, repeated stimulation of the peripheral tissue by the catheter and guidewire can lead to heart block. Additionally, the occurrence of AVB directly affects the operator's judgment of whether the operation can be performed and affects the prognosis of the patient. Therefore, this study used the WT to establish the arteriovenous loop. The catheter was inserted into the ascending aorta and aortic arch while ensuring that the catheter was not inserted into the LV. Taking advantage of the aortic valve opening, the guidewire was then inserted into the LV. The catheter was rotated and adjusted up and down to facilitate exploration of the inlet of VSD with the wire. Then, the wire drifted in the direction of flow through the VSD to the RV and pulmonary artery or through the right atrioventricular valve to the superior/inferior vena cava. In contrast to the conditions when using the CT, when using the WT, only the guidewire can affect the conduction bundle. Furthermore, we chose a loach guidewire to establish the arteriovenous loop, which produces less mechanical stimulation to the endocardium. Moreover, the WT takes advantage of hemodynamic characteristics, by which the guidewire can "drift" in the direction of blood flow; thus, the "direct violence" to the heart is also much smaller. In contrast, the traditional technique using a catheter goes through the interventricular septum. We hooked the inlet (LV surface) of the VSD directly to the catheter. However, the catheter diameter is thicker and the texture is firmer than the guidewire, which is bound to produce more stimulation to the endocardium.

The mechanisms of AVB during and after the procedure may be different. Previous studies have suggested that the main mechanisms of AVB after transcatheter closure of PmVSD were operative physical stimulation caused by defect edge edema, which affects the conduction bundle, and direct compression of the occluder on the conducting tissue. Walsh et al. concluded that AVB occurring after the procedure may be due to local inflammation and fibrosis 19. In our study, there was no significant difference in the incidence of post-procedure AVB between the two groups. Nevertheless, third-degree AVB occurred in one patient (1.9%) in the CT group within 3 days after the procedure. After the intravenous injection of dexamethasone for 7 days, the heart rhythm was still third-degree AVB. After follow-up for 3 months, the heart rhythm was still third-degree AVB, and a permanent pacemaker was implanted. The reason for this was considered as follows: LV angiography showed multiple-outlet PmVSD, and the catheter and guidewire repeatedly stimulated the surrounding tissue around the defect. When the arteriovenous loop was established, it was found that the guidewire rode across the tricuspid chordae tendineae, so the arteriovenous loop should be established again. Thus, the occurrence of AVB was associated with a longer duration of arteriovenous loop establishment; consequently, the catheter and guidewire led to irreversible damage to the atrioventricular bundle. In contrast, in the WT group, one case (1.5%) of AVB occurred after the procedure and was reverted to sinus rhythm by the intravenous injection of dexamethasone for 3 days. This result suggests that, in addition to the occluder factor, the damage to the conduction system by the catheter may be irreversible. When using the WT to establish the arteriovenous loop, even if AVB occurs after the procedure, the possibility of a transient AVB is increased.

In our study, the results confirmed that there were no statistically significant differences in the total success rate or procedure time between the two groups. However, the CT group showed a higher one-time success rate in arteriovenous loop establishment (94.3% *vs*. 91.2%). The reason for this difference is that when the maximum velocity (Vmax) of the left-to-right shunt is faster (i.e., when the pressure between the left and right ventricles is obvious), the guidewire is easier to "float" in the direction of blood flow. Therefore, there are two disadvantages of this method. First, if the defect or the pressure between the left and right ventricles is small, it is difficult for the guidewire to pass through the interventricular septum. Second, if the PmVSD has multiple outlets in the RV surface, the guidewire does not necessarily pass through the specified outlet as expected. While using the traditional method to establish the arteriovenous loop, we used the catheter to hook the inlet (LV surface) of the VSD directly. Therefore, the one-time success rate of establishing the arteriovenous loop is strongly associated with the operator; in other words, it has higher controllability. Even if the defect is small or has multiple outlets, the guidewire can pass through the interventricular septum as long as the catheter is able to hook the LV surface of the defect.

It is necessary to pay attention to certain problems when applying the WT. First, when establishing the arteriovenous loop, attention should be paid to the catheter tip position. In most cases in this study, the catheter tip was placed in the aortic arch, which provided sufficient support and force to the guidewire for it to drift directly along the catheter into the LV, through the defect, and into the RV. Second, the outlet direction of the defect determines whether the guidewire can drift successfully. In this study, difficulties were encountered in 6 patients in the WT group in drifting the guidewire through the defect successfully in one attempt because the outlets of the defect were multi-crevassed. Thus, the guidewire drifted through the minor crevasse into the RV, and the position of the guidewire consequently required readjustment to establish the arteriovenous loop. Third, the WT carries a risk of injuring a tendon. During the process of drifting the guidewire through the defect from the inlet to the outlet, the guidewire may enter a tendon and cause tendon injury. However, using the WT, regardless of whether the guidewire drifts into the tendon, as long as the guidewire travels with the direction of blood flow, it can enter the defect to access the outlet, enabling the catheter to be immediately pushed along the guidewire through the inlet and into the outlet. Then, the guidewire can be retracted into the LV and pushed along the catheter into the RV. By following the direction of blood flow, the guidewire can then drift to the distal end of the pulmonary artery or go through the right atrioventricular valve to enter the superior or inferior vena cava, which altogether can minimize the risk of tendon injury.

The results of this study demonstrate that the WT is safe and effective in pediatric patients. In certain patients, the WT could replace the traditional method of establishing the arteriovenous loop and reduce the stimulation of the peripheral tissue by the catheter and guidewire, thereby reducing the incidence of AVB during the procedure. However, for patients with small defects or multiple-outlet PmVSD, the CT is recommended.

## AUTHOR CONTRIBUTIONS

He L and Zhang YS were responsible for the conception and design. Cheng GS and Wang XY were responsible for the data analysis and interpretation. He L was responsible for the manuscript drafting. Du YJ and He XM were responsible for the critical revision of the manuscript. He L, Cheng GS, Zhang YS, He XM, Wang XY, Du YJ approved the final version of the manuscript.

## Figures and Tables

**Table 1:** Baseline Characteristics of the Two Groups.

Variable	CT Group (n=53)	WT Group (n=68)	*p*-value
Demographics			
Age, years, median (IQR)	5.0 (2.0)	4.0 (3.0)	0.085
Age group, years, n (%)			0.549
3-5	36 (67.9%)	48 (70.6%)	
6-10	11 (20.8%)	16 (23.5%)	
11-14	6 (11.3%)	4 (5.9%)	
Male, n (%)	26 (49.1%)	29 (42.6%)	0.482
Weight, kg, median (IQR)	20.0 (7.0)	18.0 (6.5)	0.104
Echocardiographic Findings			
Defect size, mm, median (IQR)	8.0 (4.0)	8.0 (1.8)	0.198
Ventricular septal rim below the aortic valve, mm, median (IQR)	3.0 (1.5)	3.0 (0.7)	0.105
MVSA, n (%)	38 (71.7%)	49 (72.1%)	0.965
Minor aortic regurgitation, n (%)	3 (5.6%)	4 (5.9%)	1.000
Minor tricuspid regurgitation, n (%)	2 (3.8%)	2 (2.9%)	1.000

MVSA, membranous ventricular septal aneurysm; IQR, interquartile range.

**Table 2:** Procedural Characteristics of the Two Groups.

Variable	CT Group (n=53)	WT Group (n=68)	*p*-value
Qp/Qs ratio, median (IQR)	2.3 (0.5)	2.1 (0.4)	0.063
sPAP, mmHg, median (IQR)	28.0±4.8	30.1±3.8	0.009
mPAP, mmHg, median (IQR)	15.0 (6.0)	18.0 (4.0)	0.004
Defect size by radiography, mm, median (IQR)	6.0 (4.3)	6.5 (5.0)	0.556
Procedure time, min, median (IQR)	46.0 (14.0)	46.5 (10.0)	0.315
Fluoroscopy time, min, median (IQR)	24.0 (8.0)	22.5 (5.0)	0.268
One-time success rate of establishing an arteriovenous loop, n (%)	50 (94.3%)	62 (91.2%)	0.730
Occluder, n (%)			0.825
Symmetric	33 (64.7%)	44 (66.7%)	
Asymmetric	18 (35.3%)	22 (33.3%)	
AVB during the procedure, n (%)	2 (3.8%)	/	
AVB after the procedure, n (%)	1 (1.9%)	1 (1.5%)	1.000
Tricuspid regurgitation post-procedure, n (%)	2 (3.8%)	/	

Qp/Qs, pulmonary-to-systemic flow ratio; sPAP, systolic pulmonary artery pressure; mPAP, mean pulmonary artery pressure; AVB, atrioventricular block; IQR, interquartile range.
